# Ethanolic extract of *Ficus religiosa* leaves alleviates aluminum-induced oxidative stress, lipid peroxidation, and neuroinflammation in rat brain

**DOI:** 10.14202/vetworld.2024.2088-2095

**Published:** 2024-09-15

**Authors:** Amit B. Massand, Ashwin R. Rai, Vandana Blossom, Mangala M. Pai, P. J. Jiji, Rajalakshmi Rai

**Affiliations:** 1 Department of Anatomy, Smt. B. K. Shah Medical Institute and Research Centre, Sumandeep Vidhyapeeth, Pipariya, Vadodara, Gujarat, India; 2 Department of Anatomy, Kasturba Medical College Mangalore, Manipal Academy of Higher Education, Manipal, Karnataka, India

**Keywords:** aluminum, brain, *Ficus religiosa*, lipid peroxidation, neurodegeneration, neuroinflammation

## Abstract

**Background and Aim:**

Aluminum (Al)-induced neurotoxicity is known to play a pivotal role in the development of various neurodegenerative diseases, and this is alleged to occur through neuroinflammation and oxidative stress in the brain. This study aimed to determine the effect of *Ficus religios*a (FR) leaf extract on oxidative stress and neuroinflammation induced by Al exposure in the rat brain by estimating malondialdehyde (MDA), interleukin-6 (IL6), and total antioxidant (TAO) levels along with the degree of neurodegeneration in the brain of AlCl_3_-administered and F*R* leaf extract-treated rats.

**Materials and Methods:**

Two- to three-month-old male albino *Wistar* rats weighing 250–280 g were used in the present study. The animals were randomly divided into seven groups, with 12 rats in each group. The groups were categorized as control, Al-intoxicated, FR treatment groups of two dosages, FR control rats of two dosages, and FR pre-treatment group.

**Results:**

We observed a substantial increase in the levels of MDA and IL6 along with a decline in the TAO level in Al-intoxicated rats, suggesting increased lipid peroxidation (LPO), neuroinflammation, and oxidative stress, respectively. In the FR-treated animals, MDA as well as IL6 levels was decreased, and TAO was enhanced in addition to improved neuronal architecture, demonstrating the ameliorative effect of FR.

**Conclusion:**

The present study observed a decline in LPO and neuroinflammation in FR-treated rats, demonstrating the protective effect of FR leaves against Al-induced neurotoxicity. The level of TAO also improved along with improvement in neuronal mass in FR-treated rats, adding to its ameliorative effect. However, further elaborate research is needed to confirm its therapeutic potential against inflammation-driven neurodegenerative diseases.

## Introduction

Aluminum (Al) is a known neurotoxin, which is presented in a concentration-dependent manner and is the most abundant element in the earth’s crust [1]. It can reach the human biological system through drinking water, food additives, utensils, body deodorants, or medicines [2]. Al is proven to be neurotoxic when inhaled or ingested beyond a certain level, and the matter of concern is that exposure to Al has increased over the years because of its necessity in the modern world, with due credit to its multifunctional quality [3]. Environmental pollution and occupational risk also increase the rate of Al exposure. Al accumulates in the hippocampus, prefrontal cortex, cerebellum, and brain stem because of prolonged exposure, particularly in the hippocampal regions, which are vulnerable to its toxicity [4, 5]. Accumulated Al in the brain generates oxidative stress that affects the nervous system structurally and functionally, leading to neurodegeneration in the form of progressive loss of neurons. It is a well-known fact that neurodegeneration is a characteristic feature of neurological diseases such as Alzheimer’s disease (AD), Parkinson’s disease, and Huntington’s disease [6, 7]. The relationship between long-term exposure to Al and AD was demonstrated in an animal model [8]. Rats intoxicated with 100 mg/kg body weight (bw) of AlCl_3_ displayed cognitive impairment and neuronal apoptosis [9].

In addition, a substantially elevated level of Al was found in patients with AD, as reported in a clinical study [10]. Furthermore, several studies in a set of populations have shown a link between Al and neurological diseases, in which cognitive loss and dementia are most prevalent [11, 12]. Usman *et al*. [5, 13] observed delayed development in the cytoarchitecture of the cerebellum and increased expression of glial fibrillary acidic protein in pups that underwent prenatal exposure to AlCl_3_.

As exposure to Al has alarmingly increased in recent years, its subsequent deleterious effect on the brain needs to be addressed seriously. In this context, the current work was carried out on AlCl_3_-administered rats to study the possible neuroprotective potential of *Ficus religiosa* (FR) leaf extract. The development of cost-effective treatment with possible minimal side effects is the need of the hour, which can treat neurodegenerative diseases in a wider population. It is a recognized fact that the antioxidant properties of plants have the healing power to protect against reactive oxygen species (ROS) generated by exposure to chemicals. This beneficial effect of plants can be attributed to their abundance of flavonoids and other polyphenols [14]. The FR is a wholesome tree with rich nutrients and phytochemicals that have been used as traditional medicine since ancient times [15]. It has antiamnesic, antibacterial, antiulcer, and antioxidant properties, which are of medicinal value [16, 17]. In a Parkinson’s rat model study, petroleum ether extract of 200 mg and 400 mg/kg bw of FR leaves improved motor deficit and oxidative damage [18], making it an ideal experimental herbal material for the present study.

This study aimed to determine the effect of FR leaf extract on oxidative stress and neuroinflammation induced by Al exposure in the rat brain. This was achieved by estimating malondialdehyde (MDA) levels for measuring the extent of lipid peroxidation (LPO), pro-inflammatory cytokine IL-6, and total antioxidants (TAO) along with the degree of neurodegeneration in AlCl_3-_administered rat brain and compared it with that of FR leaf extract-treated rats.

## Materials and Methods

### Ethical approval

The protocol of this study was approved by the Institutional Animal Ethics Committee of Kasturba Medical College, Mangalore (approval number: KMC/MNG/IAEC/06-2020). All experiments were performed according to institutional guidelines for animal experiments and ARRIVE guidelines.

### Study period and location

The study was conducted from August 2021 to July 2022 in the Central Animal House of Kasturba Medical College Mangalore.

### Animals

Eighty-four male albino *Wistar* rats aged 3–4 months (250–280 g weight) were used in this study. The rats were housed in polypropylene cages with paddy husk bedding under regular temperature (22 ± 3°C) and humidity. The rats were provided with *ad libitum* access to feed and water and the rat feed pellets were purchased from Champaka Feeds and Foods, Bangalore, India.

### Study design

The rats were randomly divided into seven groups, each with two subgroups (n = 6), one for biochemical parameters and the other for histological evaluation. The rats were acclimatized for a week before being divided into different groups. AlCl_3_ and FR leaf extract were administered through an oral gavage tube (p.o). The dosage of AlCl_3_ was 100 mg per/kg bw [9]. For FR leaf extract, two different dosages (200 mg and 300 mg/kg bw) were considered in this study because 200 mg/kg bw showed a positive effect in Parkinson’s induced rats, according to Bhangale and Acharya [18]. Treatment with FR leaf extract was for 15 days as at this duration, it exhibited a protective effect in a Huntington’s rat model [19].

### Animal groups

- Group 1: Normal control (received tap water for 45 days)
- Group 2 (Al): Al group (100 mg per/kg bw of AlCl_3_ for 4 days)
- Group 3 (T200): AlCl_3_ for 45 days followed by 200 mg/kg bw of ethanolic extract of FR leaves for 15 days
- Group 4 (T300): The animals received 100 mg/kg bw of AlCl_3_ for 45 days, followed by 300 mg/kg bw of ethanolic extract of FR leaves for 15 days
- Group 5 (FR200): Animals received 200 mg/kg bw FR leaf extract alone for 15 days
- Group 6 (FR300): Animals received 300 mg/kg bw FR leaf extract alone for 15 days
- Group 7 (PRL): Animals were pretreated with 200 mg/kg bw of FR leaf extract alone for 1 week, followed by 100 mg/kg bw of AlCl_3_ and 200 mg/kg bw FR for 45 days.

### Extraction of FR leaves

We identified and collected FR leaves from the Shobhavan Botanical Garden, Moodbidri, Karnataka. The identity of the leaves was confirmed, and a certificate of identity for FR leaves was issued by a well-known botanist, Dr. HS Shenoy, and Principal Scientist, Dr. Shivarama Karantha Pilikula Nisarga Drama, Mangalore. We excluded old, dried leaves and used medium ripe, green leaves throughout the study. Because the amount of moisture in the atmosphere was greater in this region, the leaves were allowed to dry in shade for over 6 months. When the leaves were completely dried, coarse powder was produced using a mixer grinder. Thus, the obtained coarse powder was placed in a Soxhlet apparatus containing ethanol and heated to reflux. Ethanol was used as a solvent at a ratio of 1:1 along with distilled water. Using the Soxhlet apparatus, the leaf extract of FR was prepared as described by Dhawan and Gupta [20].

### Biochemical estimation

The animals were deeply anesthetized with sodium pentobarbital (45 mg/kg bw) at the end of the 45 days in the control and Al-intoxicated groups and the next day after the last treatment (15 days of FR treatment) in the treatment groups (T200 and T300) [21]. Brain tissue was removed rapidly through cranial cavity dissection, cleaned with normal saline, and a homogenate was prepared using 0.1M PBS at pH 7.4. The homogenized sample was centrifuged (10,000 × *g*) for 20 min at 4°C, and a portion of the supernatant was collected for biochemical studies.

#### Estimation of TAO content

The TAO level in the brain homogenate was estimated using the method described by Koracevic *et al*. [22]. To each sample and its control, a Fe–ethylenediaminetetraacetic acid mixture and H_2_O_2_ were added after adding 20% acetic acid. A negative control was prepared for each analysis containing the same reagents as sample or control, except that the homogenate was replaced with phosphate buffer. For calibration, standards containing 1 mM/L uric acid were used. They were simmered in a boiling water bath at 100°C for 10 min. and immediately cooled in an ice bath. The absorbance at 532 nm against deionized water was calculated using the formula AOA (mmol/L) = (C_UA_) (K-A)/(K-UA), wherein K=absorbance of control, A=absorbance of sample, UA=absorbance of uric acid solution, and C_UA_=concentration of uric acid expressed in mM/L.

#### Estimation of MDA content

MDA was estimated in brain tissue homogenates using the method described by Rao *et al*. [23]. This method involves the reaction of MDA in the samples with thiobarbituric acid (TBA), resulting in TBA reactive substances (TBARS). In a test tube, to 0.5 mL of sample homogenate, 2.5 mL of 10% phosphotungstic acid solution was added and centrifuged at 492 × *g* for 10 min. The obtained precipitate was refrigerated at 4°C overnight. Then, 0.5 mL of TBA was added to 2 mL of supernatant, boiled for 45 min. in a water bath, and cooled immediately. Then, 0.05 mL of 5M HCl was added to 0.4 mL of this cooled solution (TBA-supernatant) and thoroughly mixed. In this mixture, 1 ml of freshly prepared solution of NaOH was added to eliminate the necessity of centrifugation. The absorbance at 535 nm wavelength was evaluated against a blank (distilled water). The volume of TBARS obtained was expressed as MDA levels, which were estimated by applying the molar extinction coefficient for MDA of 1.56 × 10^5^ cm^−1^/M. The obtained values are expressed as μmoles of MDA/g protein.

### Histological study

The next day after the last treatment with FR, the animals for histological evaluation were deeply anesthetized with sodium pentobarbital. They were perfused and fixed with 10% formalin, after which the cranial cavity was dissected, and the brain was removed. The samples were transferred to a container containing 10% formalin and processed further for histological study. Cells with disrupted cell membranes and pyknotic and hyperchromatic nuclei were considered degenerated neurons. For neuronal assays in the prefrontal cortex, cresyl violet stain was used for staining sections of brain tissue because, with this stain, the nucleus and cytoplasm of the neurons can be well differentiated with proper clarity [24].

### Statistical analysis

Statistical analysis of the obtained results was performed using a one-way analysis of variance and Tukey’s multiple comparison test. p < 0.05 was considered statistically significant. Analysis was conducted using Statistical Package for the Social Sciences software 29.0 (IBM Corp., NY, USA). All values are presented as mean ± standard deviation.

## Results

### Activity of MDA

**Figure-1:**
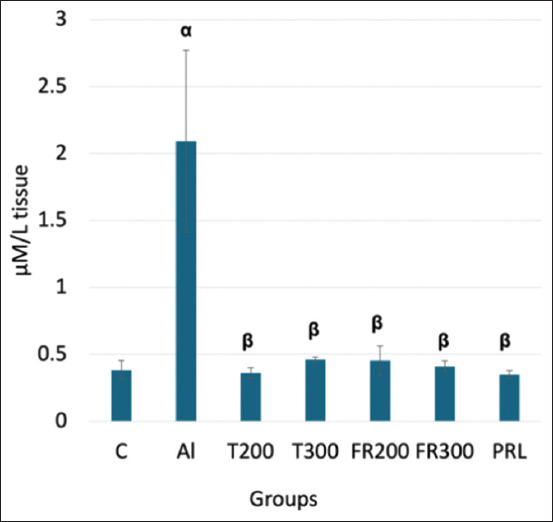
Comparison of malondialdehyde level in the brain tissue between different groups. α denotes comparison of control with Al group; β denotes comparison of Al with other groups; α-p < 0.001, β- p < 0.001 (One-way analysis of variance, Tukey *post-hoc* test (multiple comparisons), (n = 6); Values are expressed as mean ± SD; Error bar represents ±SD. SD=Standard deviation.

As shown in Figure-1, the animals in the Al group showed elevated MDA (2.09 μM/L) compared with the control (0.38 μM/L; p < 0.001). The MDA level decreased significantly in the T200 and T300 treatment groups compared with Al (0.36 and 0.46 μM/L, respectively; p < 0.001). In the FR200 and FR300 and PRL groups, we also observed significantly decreased MDA levels (0.45, 0.41, 0.35 μM/L, respectively; p < 0.001) compared with the Al group.

### Expression of TAO

**Figure-2:**
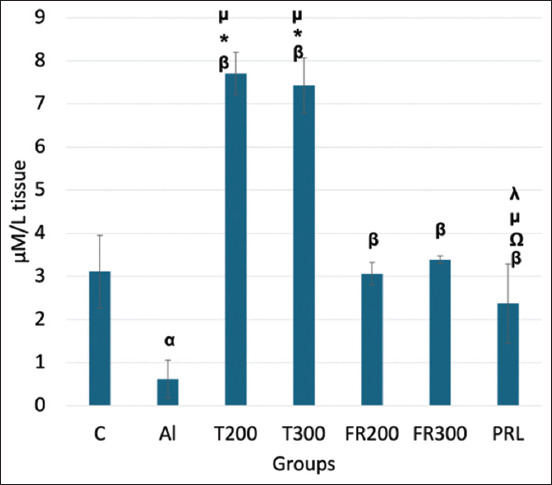
Total antioxidant level in the brain tissue of different groups. α denotes comparison of control with Al group; β denotes Al versus other groups; *-denotes FR200 versus other groups; λ denotes T200 versus other groups; Ώ denotes T300 versus other groups; μ denotes FR300 versus other groups; p < 0.001 for α, β, *, λ, Ώ and μ; (One-way analysis of variance, Tukey’s *post-hoc* test (multiple comparisons), (n = 6). Values are expressed as mean ± SD; Error bar represents ±SD. SD=Standard deviation.

As shown in Figure-2, the level of TAOs declined significantly in the Al group (0.62 mM/L; p < 0.001) than that of control (3.11 mM/L). In treated groups T200 and 300 (7.71 and 7.46 mM/L, respectively), the level of TAO elevated significantly (p < 0.001). In FR200 (3.06 mM/L), FR300 (3.39 mM/L), and PRL (2.37 mM/L) animals, TAO was significantly increased compared with Al (p < 0.001). In treated animals, the increase in the TAO level was significantly higher (p < 0.001) than that in control animals. In the PRL group, although the level of TAO increased significantly (p < 0.001) when compared to Al group, it was lower than in the treated groups (p < 0.001).

### Expression of interleukin-6 (IL6)

**Figure-3:**
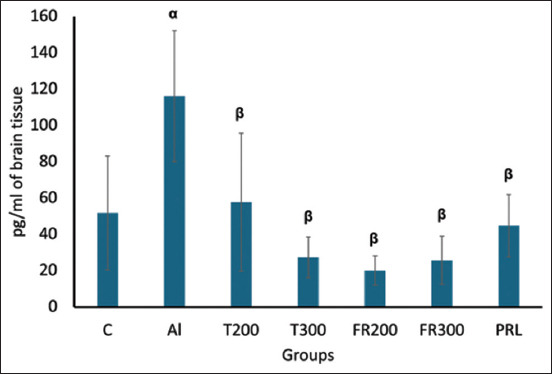
Comparison of expression of Interleukin-6 in the brain tissue between different groups. α denotes comparison of control with Al group, p < 0.001; β denotes Al versus other groups, p < 0.001; (One-way analysis of variance, Tukey’s *post-hoc* test (multiple comparisons), (n = 6). Values are expressed as mean ± SD; Error bar represents ±SD. SD=Standard deviation.

As shown in Figure-3, IL6 expression was significantly higher (116.18 pg/mL; p < 0.001) than in the control (51.86 pg/mL). In contrast, in T200 and T300, the IL6 level was significantly lower (20.03 and 25.76 pg/mL, respectively). In both groups, the expression of IL6 was much lower than that in the control animals. The FR200, FR300, and PRL animals also showed reduced IL6 levels compared with the Al group (57.76, 27.33, and 44.8 pg/mL, respectively; p < 0.001).

### Histological evaluation

**Figure-4:**
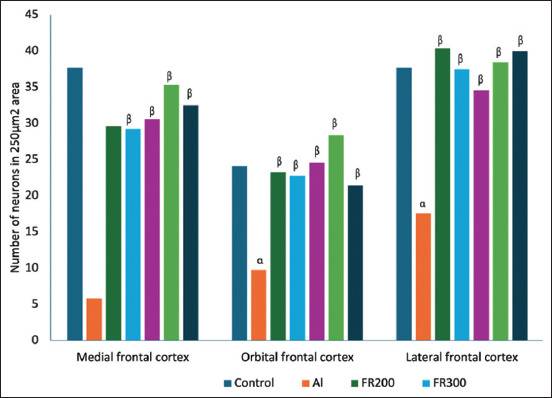
Number of viable neurons in the prefrontal cortex (medial, orbital, and lateral). α denotes the comparison of control with Al group; β denotes comparison of Al with other groups. α – p < 0.001, β – p < 0.001. (One-way analysis of variance, Tukey multiple comparison test, n = 6 in all groups). Values are expressed as mean values (n = 6).

**Figure-5:**
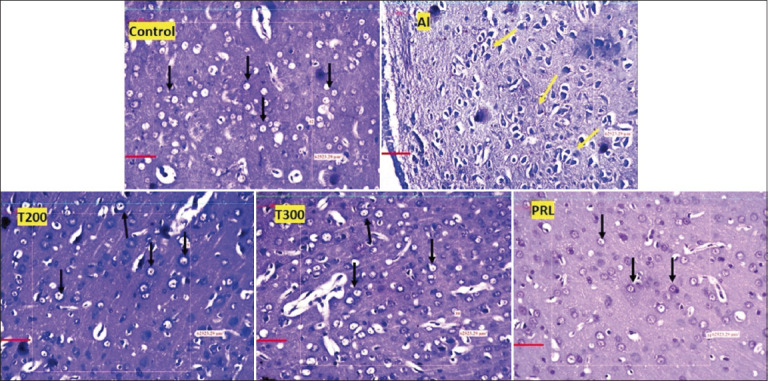
Photomicrographs of the prefrontal cortex in different groups (20×). Black arrow, viable neurons; yellow arrow, degenerated neurons; stain, Cresyl violet; Scale bar - 50 μ.

In the neuronal assay, viable neurons were counted in 250 μm^2^ area of medial, lateral, and orbital areas of the prefrontal cortex of brain tissue. In the animals of the Al group, neuronal loss was visible in the form of a significant decline (p < 0.001) in the number of viable neurons compared with the control group. In the treated groups (T200 and T300), the FR200 and 300 groups, as well as the PRL group, there was a significant (p < 0.001) enhancement in the number of viable neurons compared with the Al group (Figures-4 and 5), indicating improvement after treatment. In the medial and orbital prefrontal cortex, the highest numbers of neurons were observed in the T300 group compared with the control group. In the lateral prefrontal cortex, the highest number of neurons was observed in the FR200 and PRL groups after the control group.

## Discussion

Al is a known neurotoxin, and its chronic exposure in different forms generates oxidative stress and LPO, which eventually leads to neurodegeneration in the brain [25]. Due to its high metabolic activity, the brain is more vulnerable to ROS formation, and long-term exposure to Al increases ROS production. When the balance between the production of pro-oxidants and antioxidants in a cell or tissue is disturbed, redox signaling is disrupted, and free radicals lead to oxidative stress [26, 27]. The depth of oxidative stress can be measured in tissues by estimating the levels of MDA, antioxidant enzymes, and TAOs.

In the present study, neuroinflammation and neurodegeneration induced by the AlCl_3_ were attenuated by FR leaf extract treatment in rats. This was evident in the form of a reduction in MDA and IL6 levels and an increase in TAO levels in the treatment groups (T200 and T300). The cytoarchitecture of the frontal cortex also improved after FR administration. Improvements in biochemical parameters were linked with improvements in neuronal structure and frontal cortex density in FR-treated rats. In addition, it was observed that among the two dosages of FR leaf extract, 300 mg/kg bw produced better results than 200 mg.

Increased LPO is a major event resulting from oxidative stress, whereby oxygen reacts with unsaturated lipids to generate oxidation products that destroy the cell membrane [28]. There are two major by-products of LPO: MDA and 4-hydroxy-2-nonenal [29]. MDA is a commonly used biomarker for estimating the extent of LPO. During this process, free radicals attack lipids containing double bonds of carbon [30]. They stop the chain reaction by directly reacting with peroxide, leading to oxidative damage to the cell membrane through LPO. However, LPO can promote cell survival or induce apoptosis under specific metabolic conditions. Low or optimal rates of LPO stimulate the survival and maintenance of cells through antioxidant defense mechanisms or specific signaling pathways.

In contrast, a high level of LPO overrides the repair capacity, inducing programmed cell death. The secondary end product of LPO, MDA, acts as a signaling messenger during the process, and excessive MDA levels lead to neurodegeneration [31]. Such neurodegeneration was evident in AlCl_3_-injected rats where the MDA was significantly increased_,_ and they exhibited senile dementia similar to AD [32]. In another study that was carried out to quantify the expression of MDA by HPLC, MDA expression was significantly increased in AlCl_3_-exposed rat brains [33]. In agreement with the aforementioned reports, we have witnessed a substantial increase in MDA content in Al-exposed rat brains (Figure-1), suggesting increased LPO. The level of MDA substantially decreased in the FR-treated rats in the present study. This suggests the antioxidant potential of FR leaf extract, which is in accordance with the results of Bhangale *et al*. [19], in which the petroleum ether extract of FR leaves was shown to reduce MDA content in a Huntington disease animal model. TAO levels decreased in the brain tissue of Al-exposed rats compared with control rats in our study. This suggests that Al toxicity can weaken the activity of the antioxidant defense system. A similar result was observed by Khalil *et al*. [34] and Ghadigaonkar *et al*. [35] when rats were treated with AlCl_3_ for 60 days, during which the TAO level decreased. The aqueous extract of FR leaves has demonstrated free radical-scavenging activity through their phenolic and flavonoid contents [36]. In agreement with this, in the present study, FR leaf extract exhibited antioxidant properties in the form of substantially enhanced TAO activity in the brains of AlCl_3_-exposed rats (Figure-2). The inflammatory process is a body defense mechanism to protect against tissue injury due to infections and certain chemical or physical stimuli, and in turn, initiates the healing process [37, 38]. During this process, ROS, NO, IL6, tumor necrosis factor-α (TNF-α), iNOS is produced [39, 40]. However, some of these cytokines may produce certain pathological changes at excessive levels [41]. One such cytokine is IL-6, which acts like a double-edged sword [42]. Circulating IL6 levels are steadily low or optimal under normal conditions. It is opined that IL-6 influences astrocytes, inhibits TNF-α-induced vascular cell adhesion molecule-1 upregulation in primary astrocytes, and functions as an anti-inflammatory cytokine [43]. When they are excessively produced, IL6 stimulates the target cell through the membrane-bound interleukin 6 receptor, which binds with the gp130 signaling receptor protein, which then activates the mitogen-activated protein kinase (MAPK) pathway [44]. This pathway deals with normal physiological function or pathological changes that regulate the survival or apoptosis of cells. The level of IL6 was increased in the blood after the administration of Al sulfate in rats, suggesting that Al toxicity triggers inflammation [45]. Likewise, in AlCl_3_-exposed rats, the level of antioxidants decreased and pro-inflammatory cytokines like TNF-α and IL6 increased, implying Al-associated neuroinflammation [12]. In the present study, we observed substantially increased levels of IL6 in the brain after administering of AlCl_3_ than that of control animals (Figure-3) and we have observed degenerative changes in the frontal cortex of Al-intoxicated rats (Figures-4 and 5). This increase might result from the rat’s immune response to Al toxicity. FR leaf extract treatment significantly reduced the level of IL6 in the rat brain, supporting its anti-neuroinflammatory potential against Al-induced inflammation. A similar anti-inflammatory response to FR leaf extract was exhibited in LPS-stimulated microglial cell lines in mice [46]. The inhibition of IL-1 and 6 levels by FR leaf extract was attributed to the downregulation of MAPK pathways.

Al-induced neurotoxicity leading to degenerative disorders in the central nervous system has been well-established in previous studies by Fernandes *et al*. [47] and Kahn *et al*. [48]. Tripathi *et al*. [46] observed neurodegeneration in the frontal cortex in the form of vacuolization in neurons, spongiform lipofuscin, and lysosomal degradation, and reduced synapses in the frontal cortex after exposure to 100 mg/kg/bw AlCl_3_ for 90 days. The present study agrees with the above reports, where we have administered the same dosage of AlCl_3_ (100 mg/kg/bw) for 45 days and observed neuronal degeneration in the prefrontal cortex in the form of disrupted cell membrane and vacuoles inside the neurons (Figure-5). In neurodegenerative disorders like AD, neuronal damage observed in the hippocampus and temporal lobes was initially restricted to the frontal lobe [49]. Because the medial and orbital frontal cortices receive direct input from the hippocampus, any lesion in these regions of the frontal cortex can also be a causative factor for neurological disorders like AD [50]. In our study, we observed neuronal loss in all three areas, that is, medial, lateral, and orbital prefrontal areas. However, when cells were treated with FR leaf extract, the number of viable neurons increased, and the cytoarchitecture was improved in all three areas of the prefrontal cortex (Figures-4 and 5).

## Conclusion

The findings of this study implicate that FR leaf extract has protective effects against neuroinflammation and oxidative stress in the rat brain. This was evident in the form of a decrease in MDA and IL6 levels in the FR-treated rat brain, which was increased by AlCl_3_ exposure. This result corresponded with the enhanced TAO levels and improved neuronal architecture in the FR-treated rats. These results add to the existing data on the protective role of FR leaves in combating neurodegeneration, which is driven by neuroinflammation and oxidative stress.

However, this study’s limitation was that other inflammatory markers like TNF-α and interleukins like IL-1β were not included. This study opens avenues for further research with larger samples and other inflammatory markers, preferably using a transgenic animal model to confirm the neuroprotective role of FR leaves.

## Authors’ Contributions

AMB: Study design, data collection, analysis, interpretation, and drafted the manuscript. ARR: Study design, supervision, and data analysis and interpretation. VB: Data collection and statistical analysis. MMP and PJJ: Data analysis and interpretation of the results and edited the manuscript. RR: Conception, supervision, study design, data interpretation, and drafted the manuscript. All authors have read and approved the final manuscript.
